# Pulmonary hypertension caused by fibrosing mediastinitis: A case report and literature review

**DOI:** 10.1097/MD.0000000000046180

**Published:** 2026-01-02

**Authors:** Kaiyue Yue, Xiaoyan Qu, Famiao Zhang, Mingdong Zhao

**Affiliations:** a Department of Respiratory and Critical Care Medicine, The Second People’s Hospital of China Three Gorges University, Yichang, China.

**Keywords:** atelectasis, fibrosing mediastinitis, hemoptysis, pulmonary hypertension

## Abstract

**Rationale:**

Fibrosing mediastinitis (FM) is a rare benign disorder characterized by excessive fibrotic proliferation within the mediastinum. Due to its insidious onset and nonspecific clinical presentation, FM is often misdiagnosed or diagnosed at a late stage, leading to significant morbidity. Case reports detailing its long-term course and response to interventional management remain limited. This case is reported to highlight the diagnostic challenges in FM presenting with prolonged hemoptysis and to demonstrate the therapeutic potential of pulmonary vascular intervention in alleviating vascular compression and improving hemodynamics.

**Patient concerns:**

A 56-year-old male patient presented with intermittent hemoptysis lasting over 5 years, which had worsened over the past week.

**Diagnoses:**

Imaging studies, including contrast-enhanced chest computed tomography and pulmonary artery CTA, revealed calcified mediastinal lymph nodes in both hilar regions, right middle lobe atelectasis, and pulmonary artery branch stenosis with secondary pulmonary hypertension. A final diagnosis of fibrosing mediastinitis complicated by pulmonary hypertension and lobar collapse was established.

**Interventions:**

Five years earlier, the patient underwent bronchoscopy and received cryotherapy and dilation for right middle lobe atelectasis, with no significant improvement observed. During the current admission, right heart catheterization and pulmonary angiography revealed multiple sites of extrinsic pulmonary artery stenosis, for which stents were implanted at the narrowed segments.

**Outcomes:**

Following the procedure, pulmonary artery pressure decreased significantly, clinical symptoms improved markedly, and no further episodes of hemoptysis were reported during follow-up.

**Lessons:**

This case underscores the importance of considering FM in the differential diagnosis of chronic unexplained hemoptysis, particularly when accompanied by mediastinal calcification, lobar collapse, and pulmonary hypertension. Early use of pulmonary artery CTA can aid in timely diagnosis, and endovascular stent placement represents a effective minimally invasive treatment option for selected cases of FM-induced pulmonary artery stenosis.

## 1. Introduction

Fibrosing mediastinitis (FM), also known as sclerosing mediastinitis or mediastinal fibrosis, is a rare benign and nonneoplastic disorder of the mediastinum characterized by excessive proliferation of fibrous tissue within the mediastinum. The proliferative fibrotic tissue often invades pulmonary vasculature, leading to vascular stenosis or occlusion, which may subsequently result in pulmonary hypertension and right heart failure (one of the major causes of mortality in FM). FM typically has an insidious onset and nonspecific clinical symptoms, with variable manifestations depending on the location and extent of mediastinal compression, making it prone to misdiagnosis or underdiagnosis in clinical practice. This article presents a rare case of FM manifesting primarily as intermittent hemoptysis, and provides a review of the relevant literature to enhance clinicians’ understanding of the disease, summarize its clinical features and management experience, and offer insights for future diagnosis and treatment.

## 2. Case presentation

### 2.1. General information

A 56-year-old male presented to our hospital on January 11, 2023, with a history of intermittent hemoptysis lasting >5 years, which had worsened over the past week. The patient reported that the hemoptysis began 5 years ago without any obvious trigger. The volume was approximately 5 mL, bright red in color, and not accompanied by low-grade fever, night sweats, chest tightness, chest pain, cough, or sputum production. He was previously evaluated at a hospital in Wuhan, where he was diagnosed with right middle lobe atelectasis and underwent 2 sessions of bronchoscopic cryotherapy and dilation (details unavailable), but the atelectasis did not resolve. Six months ago, he experienced another episode of hemoptysis. A chest computed tomography (CT) performed at a local hospital revealed pulmonary artery dilation, right middle lobe atelectasis, and bilateral pleural effusion. He was discharged after symptomatic treatment (specific regimen unknown). One week prior to admission, he developed a cough with blood-streaked sputum without an identifiable cause. He denied purulent sputum, fever, chest tightness, chest pain, nausea, or vomiting. On January 7, 2023, he visited a hospital in Enshi, where he was initially diagnosed with bilateral bronchiolitis. He was prescribed oral levofloxacin and Yunnan Baiyao capsules, which led to mild symptom relief. To clarify the diagnosis, he presented to our hospital on January 11, 2023, and was admitted to the Department of Respiratory Medicine with a provisional diagnosis of “hemoptysis of unknown cause.” Since the onset of symptoms, his mental status, appetite, and sleep remained stable, though he reported mild fatigue. No significant weight change was noted, and bowel and bladder functions were normal.

*Past medical history*: He denied a history of hypertension, diabetes, coronary artery disease, or infectious diseases such as hepatitis and tuberculosis. He previously underwent bronchoscopic cryotherapy and dilation for right middle lobe atelectasis 5 years ago. No history of drug allergy or trauma was reported.

*Personal and family history*: He had a 4-year history of working in coal mining. He smoked approximately 20 cigarettes per day for 20 years and quit smoking 10 years ago. He denied any familial history of hereditary disorders or malignancies.

*Physical examination*: Temperature 36.5 °C, pulse 60 bpm, respiratory rate 21 breaths/min, blood pressure 106/65 mm Hg. He was alert and oriented, with no cyanosis of the lips and no palpable or tender superficial lymph nodes. Breath sounds were diminished over the right lung field, with no rales or rhonchi detected. Cardiac auscultation revealed a regular rhythm and normal heart sounds without murmurs over the valvular areas. The abdomen was flat and soft, with no tenderness or rebound tenderness. The bladder was not distended, and no renal masses were palpable. There was no costovertebral angle tenderness. No edema was observed in either lower limb.

### 2.2. Investigations

#### 2.2.1. Laboratory tests

A complete blood count on January 12, 2023, showed: white blood cell count 4.2 × 10⁹/L, neutrophil count 2.1 × 10⁹/L, lymphocyte count 1.7 × 10⁹/L, hemoglobin 136 g/L, and platelet count 217 × 10⁹/L. Liver function tests revealed total protein of 66.2 g/L and albumin of 38.7 g/L. Renal function results showed a blood urea nitrogen level of 8.23 mmol/L and serum creatinine of 98 μmol/L. Arterial blood gas analysis (at rest with conventional oxygen inhalation) indicated: pH 7.44, PaO₂ 130 mm Hg, PaCO₂ 38 mm Hg, oxygen saturation 99%, and an oxygenation index of 619. Tumor markers (including pro-gastrin-releasing peptide, cytokeratin-19 fragment, carcinoembryonic antigen, neuron-specific enolase, and squamous cell carcinoma antigen) were all within normal limits. D-dimer, cardiac enzymes, myocardial infarction biomarkers, coagulation parameters, B-type natriuretic peptide, procalcitonin, interleukin-6, C-reactive protein, and sputum cultures were all unremarkable.

#### 2.2.2. Imaging and specialized examinations

**Figure 1.**
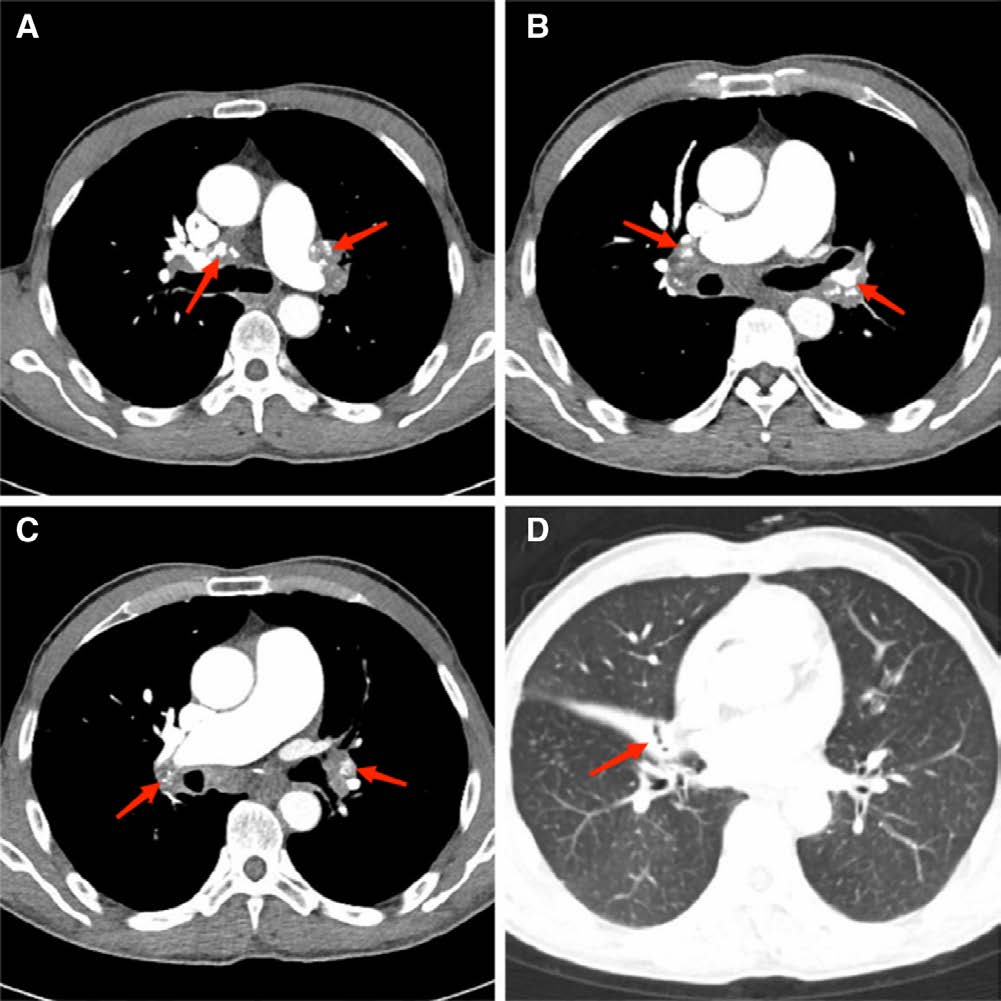
Contrast-enhanced chest CT findings upon admission. (A–C) Bilateral hilar and mediastinal lymph nodes show heterogeneous enlargement with poorly defined margins, and partial calcification is observed (red arrows). (D) Atelectasis of the right middle lung lobe is evident (red arrow). CT = computed tomography.

**Figure 2.**
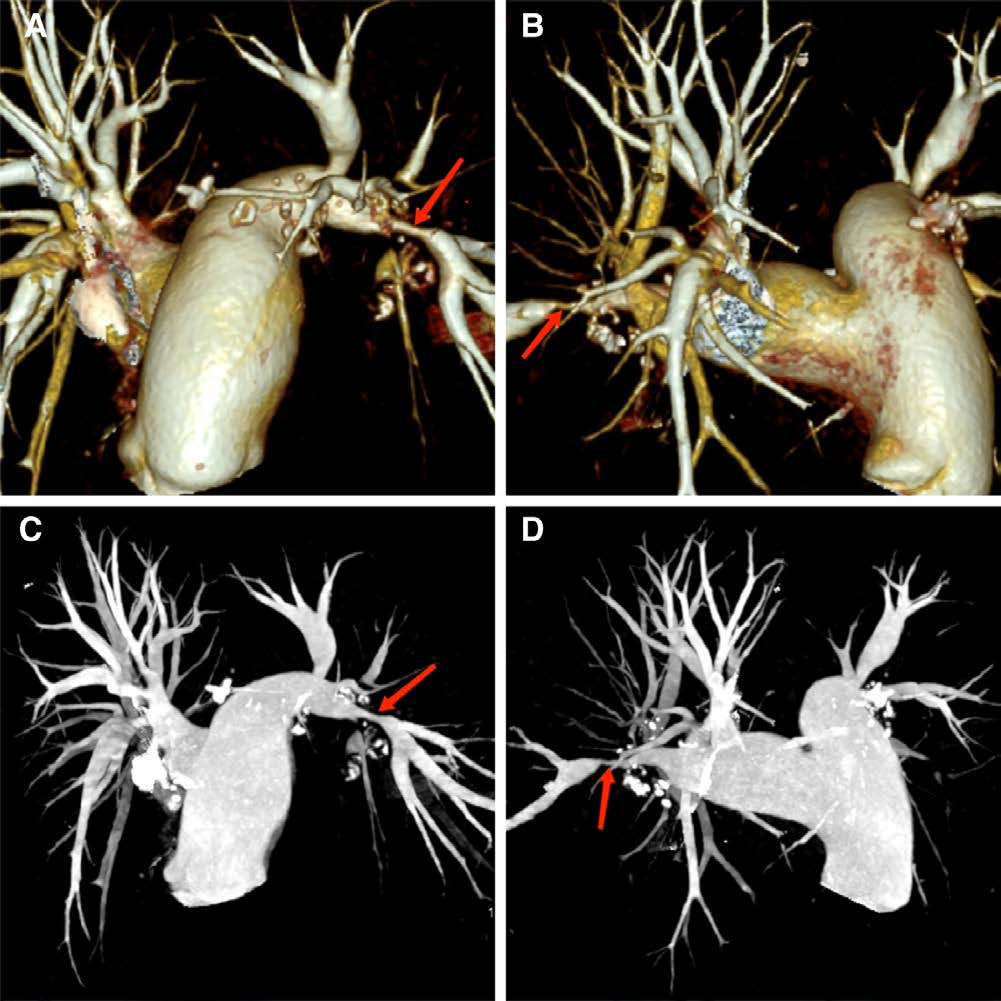
Pulmonary artery CTA findings upon admission. (A and C) Compression and narrowing of the right pulmonary artery branches. (B and D) Compression and narrowing of the left pulmonary artery branches.

On January 7, 2023, chest CT from an outside hospital revealed bilateral bronchiolitis, right middle lobe atelectasis, thickening of the pulmonary artery trunk, and enlarged hilar and mediastinal lymph nodes with partial calcification. On January 12, transthoracic echocardiography demonstrated right heart enlargement and dilation of the pulmonary artery (diameter ~37 mm), consistent with severe pulmonary hypertension; the estimated pulmonary artery pressure was 80 mm Hg. Mild mitral and tricuspid regurgitation were also observed, along with reduced left ventricular diastolic function. Contrast-enhanced chest CT combined with pulmonary artery CTA and bronchial artery CTA (Figs. 1 and 2) showed sparse and disorganized pulmonary markings with increased lung translucency. Diffuse high-density nodules were observed in both lungs, and multiple calcified lymph nodes were noted in the bilateral hilar regions. The branches of the pulmonary arteries appeared compressed and narrowed, while the main pulmonary artery was thickened, measuring approximately 37 mm in diameter; the ascending aorta measured about 33 mm at the same level. The distal branches of the pulmonary arteries demonstrated smooth courses without significant filling defects. The aorta and its major branches appeared patent, with no obvious signs of stenosis or aneurysmal dilatation. The right bronchial artery was mildly dilated and originated from the anterior wall of the thoracic aorta at the T6 vertebral level, while the left bronchial artery was not visualized. Based on these findings, the following were considered: bilateral hilar pulmonary artery branch compression and narrowing, pulmonary hypertension, aortic atherosclerosis, mild dilation of the right bronchial artery, and signs of chronic bronchitis and emphysema with diffusely distributed micronodules in both lungs, for which an occupational exposure history should be considered. Pulmonary function testing showed an FEV_1_/FVC ratio of 74.99% and an FEV_1_ of 104.20% of predicted. After bronchodilator inhalation, the FEV_1_/FVC increased to 93.20%, and the bronchodilator test was negative, indicating mild obstructive ventilatory impairment.

### 2.3. Diagnosis and differential diagnosis

Based on the patient’s clinical manifestations and auxiliary examinations, the final diagnoses were: fibrosing mediastinitis, acquired pulmonary artery stenosis with pulmonary hypertension, and right middle lobe atelectasis. Differential diagnoses included the following: *Pulmonary embolism*: clinically characterized by dyspnea, chest pain, hemoptysis, and syncope. Chronic pulmonary embolism may result in unresolved and organized thrombi, leading to pulmonary artery stenosis and progressive pulmonary hypertension. Elevated D-dimer levels and intraluminal filling defects visible on CT pulmonary angiography support the diagnosis. *Sarcoidosis*: a systemic granulomatous disease defined by noncaseating epithelioid cell granulomas, most frequently involving the mediastinal and hilar lymph nodes. Radiological findings often include mediastinal and hilar lymphadenopathy, along with diffusely distributed micronodules aligned along vascular and bronchial bundles. *Mediastinal-type lung cancer*: commonly observed in central-type lung cancer with accompanying atelectasis. The lesion typically forms an irregular soft tissue mass adjacent to the mediastinum, potentially invading the pulmonary arteries and veins, and resulting in mediastinal and hilar lymphadenopathy. Contrast-enhanced CT may reveal bronchial narrowing or occlusion with irregularly enhanced nodules or masses.

### 2.4. Treatment

**Figure 3.**
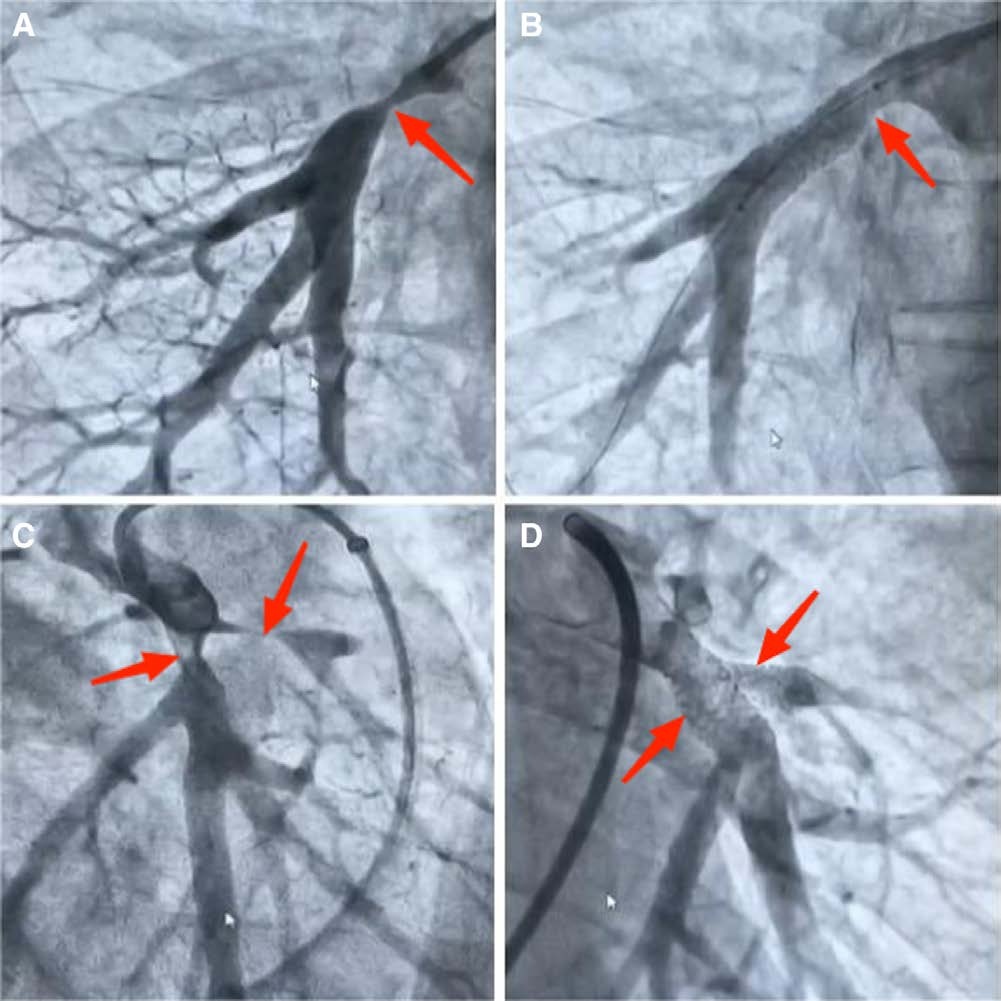
Pulmonary angiography and stent implantation performed at an outside hospital. (A) Significant stenosis (red arrow) of the right pulmonary artery branch before stent implantation. (B) Right pulmonary artery branch after stent implantation. (C) Significant stenosis (red arrow) of the left pulmonary artery branch before stent implantation. (D) “Y-shaped” stent implanted in the left pulmonary artery branch.

The patient had a long-standing history of intermittent hemoptysis and was previously diagnosed at an outside hospital with right middle lobe atelectasis. He underwent bronchoscopic evaluation and received cryotherapy and dilation; however, the lobe failed to re-expand and his symptoms did not improve significantly. At our hospital, contrast-enhanced chest CT and pulmonary artery CTA revealed multiple calcified lymph nodes in both hila, compression-induced stenosis of pulmonary artery branches, thickening of the pulmonary artery trunk, and patent distal branches without evident filling defects. These imaging findings were consistent with fibrous connective tissue proliferation compressing mediastinal structures, leading to airway and vascular narrowing, which subsequently caused pulmonary artery stenosis, pulmonary hypertension, and atelectasis. This provided a reasonable explanation for the patient’s long-term intermittent hemoptysis. After thorough discussion with the patient and his family, pulmonary vascular intervention was recommended to relieve the stenosed segments. The patient was transferred the following day to a hospital in Wuhan, where he underwent pulmonary artery stent implantation. Postoperative evaluation demonstrated a significant decrease in pulmonary artery pressure (Fig. 3).

### 2.5. Outcome and follow-up

Right heart catheterization and pulmonary angiography at the referral hospital confirmed multiple areas of pulmonary artery stenosis due to external compression. Two stents were implanted in the stenotic regions. One-month postoperative echocardiography revealed right heart enlargement and dilation of the pulmonary artery (diameter 32 mm), along with moderate pulmonary hypertension (estimated pulmonary artery pressure 55 mm Hg). This revealed a marked reduction in pulmonary artery pressure. The patient experienced significant symptomatic improvement and did not experience recurrent hemoptysis during follow-up.

## 3. Discussion

FM is a rare mediastinal disorder characterized by abnormal fibrous tissue proliferation within the mediastinum and is classified into granulomatous and non-granulomatous types.^[1]^ The granulomatous form is more common and often occurs secondary to infections with *Histoplasma capsulatum* or *Mycobacterium tuberculosis*.^[2,3]^ The etiology of FM is multifactorial, involving infectious, autoimmune, and idiopathic mechanisms. Studies have shown geographic variation in causation: tuberculosis is the predominant etiology in China,^[4]^ while histoplasmosis is more frequently implicated in Western countries.^[5]^ The precise pathogenesis of FM remains unclear; however, chronic inflammation triggered by the host’s immune response to persistent antigens is considered a likely mechanism leading to progressive fibrotic proliferation.^[6]^ In the present case, the underlying etiology could not be definitively identified due to incomplete testing resulting from financial constraints.

Clinically, FM is often nonspecific, with symptoms largely depending on the anatomical site and degree of mediastinal compression.^[4]^ Commonly affected structures include pulmonary vessels, the superior vena cava, airways, and esophagus. Compression of the pulmonary vasculature may cause dyspnea, chest pain, and hemoptysis; involvement of the superior vena cava can manifest as dizziness, headache, and swelling of the face, neck, and upper limbs; airway compression may lead to dyspnea, cough, atelectasis, and obstructive pneumonia; and esophageal compression may cause dysphagia or chest pain. Involvement of the pulmonary vessels resulting in vascular stenosis or occlusion, followed by pulmonary hypertension and right heart failure, is one of the most common fatal complications of FM. Importantly, FM with pulmonary hypertension often has an insidious onset and lacks specific symptoms, making early diagnosis difficult and increasing the likelihood of misdiagnosis or delayed recognition.^[7]^ Prompt identification is essential to improve patient outcomes. In the present case, the patient experienced long-standing intermittent hemoptysis as the primary symptom, with a gradually progressive clinical course. Imaging suggested pulmonary hypertension and right middle lobe atelectasis, while pulmonary function testing indicated mild obstructive ventilatory dysfunction. These features can mimic other causes of intermittent hemoptysis, thereby increasing the risk of diagnostic error.

Currently, there is no standardized diagnostic criterion for FM. Although histopathological examination is considered the gold standard for definitive diagnosis, its clinical application is limited due to high procedural risk and difficulty in obtaining deep mediastinal tissue samples. Consequently, the diagnosis of FM primarily relies on imaging findings. Contrast-enhanced chest CT plays a critical role in assessing the extent of mediastinal soft tissue infiltration, the degree of calcification, and the location and severity of vascular and airway stenosis.^[8]^ Radiologically, FM typically presents in 2 patterns on CT: the focal type appears as a localized soft tissue mass, often situated adjacent to the trachea, below the carina, or in the hilar region, and tends to compress adjacent vessels or airways, resulting in stenosis or obstruction. The diffuse type appears as ill-defined infiltrative lesions without a clear capsule and rarely shows cystic changes or necrosis. On contrast-enhanced CT, it typically demonstrates moderate and progressive enhancement.^[9]^ Additional CT findings may include atelectasis, mosaic perfusion pattern, ground-glass opacities, and interlobular septal thickening.^[10]^ Therefore, for patients clinically suspected of having FM, contrast-enhanced chest CT should be the initial imaging modality of choice. If severe complications such as pulmonary hypertension are present, early performance of CT pulmonary angiography is recommended to more accurately assess the degree of pulmonary vascular stenosis, rule out other chronic obstructive pulmonary vascular diseases, and guide subsequent therapeutic decisions. In the present case, imaging findings were highly consistent with FM, including heterogeneously enlarged mediastinal lymph nodes with partial calcification and ill-defined margins; thickening of the main pulmonary artery with compression-induced narrowing of the distal branches; and fibrous thickening and structural changes in the hilar and mediastinal regions, suggesting significant fibrous tissue proliferation.

There is currently no universally accepted expert consensus on the treatment of FM, and management strategies should be individualized based on clinical symptoms, the involved mediastinal structures, and the severity of involvement.^[6,10]^ Available therapeutic options include pharmacologic therapy, vascular interventional procedures, and surgical intervention. For infectious FM with confirmed etiology, systemic antifungal therapy is appropriate in cases of histoplasmosis, whereas tuberculosis-associated FM should be treated with standard antituberculosis regimens. For FM secondary to specific diseases such as sarcoidosis or IgG4-related disease, corticosteroids may be considered. Rituximab has also been reported to relieve symptoms in selected patients.^[11]^ In cases unresponsive to medical therapy, surgical decompression of affected mediastinal structures may be evaluated. For patients with concomitant pulmonary hypertension, percutaneous pulmonary vascular intervention (e.g., stent implantation) is one of the most effective options, capable of significantly alleviating symptoms, slowing disease progression, and even improving prognosis.^[12]^ In the present case, the patient underwent pulmonary artery stent implantation following definitive diagnosis. Postoperatively, pulmonary artery pressure decreased significantly, with marked symptomatic improvement.

In summary, FM is a rare benign disorder characterized by nonspecific clinical manifestations, frequently resulting in misdiagnosis as chronic bronchitis, pulmonary embolism, or lung cancer. The present case, involving long-term recurrent hemoptysis accompanied by imaging features such as mediastinal calcification, pulmonary artery stenosis, and atelectasis, highlights the importance of including FM in the differential diagnosis of unexplained hemoptysis with concurrent pulmonary hypertension. Given the challenges and high risks associated with pathological biopsy, the diagnosis of FM relies predominantly on imaging evaluation. CT and CTA play pivotal roles in delineating the extent of mediastinal fibrosis, the severity of vascular stenosis, and associated complications. Regarding management, the patient in this case underwent pulmonary artery stent implantation, which led to a significant reduction in pulmonary artery pressure and considerable symptomatic improvement. This outcome supports the role of endovascular intervention as an effective therapeutic strategy for alleviating FM-induced pulmonary artery stenosis, particularly when pharmacological or surgical options are unsuitable or ineffective. However, the etiology of FM remains complex and multifactorial, involving infectious, immune, and idiopathic mechanisms. Currently, there is no unified classification or consensus regarding its pathogenesis. Moreover, the insidious onset and nonspecific symptoms of FM often contribute to diagnostic delays, as exemplified by the 5-year interval from symptom onset to definitive diagnosis in this case, underscoring the persistent challenges in early clinical detection. Existing pharmacological approaches (including antifungals, antituberculous agents, or corticosteroids) are effective only in a subset of patients, while surgical interventions are associated with substantial risks. Although interventional therapies show promising potential, they require advanced technical expertise and are limited by specific indications. Furthermore, due to the rarity of FM, large-scale studies investigating its long-term natural history, treatment outcomes, and prognosis are lacking. Therefore, further research is urgently warranted to establish standardized diagnostic and therapeutic protocols.

## Author contributions

**Writing – original draft:** Kaiyue Yue, Xiaoyan Qu.

**Writing – review & editing:** Famiao Zhang, Mingdong Zhao.
